# Huatan Dingji Decoction intervening in atrial fibrillation: protocol for a randomized double-blind single-simulated placebo-controlled clinical trial

**DOI:** 10.1186/s13063-021-05522-z

**Published:** 2021-10-11

**Authors:** Ying Xiao, Xinyi Wang, Jin Yang, Keke Liu, Mianmian Li, Zilin Ma, Bing Deng, Lin Shen, Yihong Wei, Shuai Zhang, Na Zhang, Ping Zhao, Chen Zhu, Meijiao Mao, Nuo Tang, Qiong Wu

**Affiliations:** 1 Department of Cardiology, Longhua Hospital, Shanghai University of Traditional Chinese Medicine, 725 Wanping South Road, Shanghai, 200032 China; 2 Shanghai University of Traditional Chinese Medicine, Shanghai, 201203 China

**Keywords:** Atrial fibrillation, Traditional Chinese Medicine, Huatan Dingji Decoction, Randomization double-blind single simulation

## Abstract

**Background:**

Atrial fibrillation (AF) is one of the most common cardiac arrhythmias and can lead to heart failure (HF), stroke, pulmonary embolism (PE), and other complications, seriously affecting people’s quality of life and health. Western medicine is limited in the treatment of AF, while Traditional Chinese Medicine (TCM) has unique advantages, such as less side effects, low toxicity, long effect duration, and high compliance. The prescription of HTDJ is a common prescription for the treatment of atrial fibrillation in Longhua Hospital, Shanghai University of Traditional Chinese Medicine. It has been used for many years and has a large number of clinically effective cases. It has a good clinical application prospect, but there is a lack of effective evaluation of its clinical efficacy.

**Method:**

This study adopts a randomized double-blind, single-simulated, placebo-controlled research method. Participants were randomly assigned in a 1:1 ratio through a centrally controlled, computer-generated, simple randomization schedule. Participants would take the medicine for 1 month, and the curative effect would be evaluated. Subsequently, the participants would not take TCM and only receive western medicine treatment. They would be followed up for another 8 weeks, and a clinical evaluation would be conducted. The secondary outcomes include echocardiography, Hamilton Anxiety Scale, Hamilton Depression Scale, rate of increase and decrease of anti-arrhythmia western medicine, the MOS 36-item short-form health survey, N-terminal-pro hormone B-type natriuretic peptide level, and integral TCM syndrome score. Adverse events will be monitored throughout the trial. Cases are from outpatient and inpatient with atrial fibrillation in the Cardiology Department of Longhua Hospital. Evaluations will be conducted at baseline and at weeks 4 and 12 after randomization.

**Discussion:**

In this study, the efficacy and safety of HTDJ plus western medicine in the treatment of atrial fibrillation (qi deficiency and phlegm opacities) will be evaluated, so as to provide medical evidence of short-term and medium-term clinical efficacy for the treatment of atrial fibrillation with integrated traditional and western medicine and lay a foundation for further clinical development and application.

**Trial registration:**

ClinicalTrials.govChiCTR2000030517. Registered on March 5, 2020, with the Chinese Clinical Trial Registry

## Introduction

Atrial fibrillation (AF) is a kind of supraventricular tachyarrhythmia characterized by rapid and disordered atrial electrical activity. It is one of the most common arrhythmias. It is estimated that approximately 2% of the global population has AF, and the incidence is increasing year by year, with a fivefold increase expected in the next 40 years [1]. AF can aggravate heart failure, increase the risk of ischemic stroke and malignant arrhythmia events, and increase the incidence with age, changes in heart structure and function, a variety of underlying diseases, bad living habits, etc., leading to increased hospitalization days, medical expenses, and overall mortality [2].

The current western medicine treatment for AF mainly includes drug therapy, left atrial appendage closure (LAAC), and radiofrequency ablation (RF). Pharmacotherapy, including cardioconversion of sinus rhythm and control of ventricular rate, should be used in combination with anticoagulant therapy. However, in the course of drug treatment of AF, most of the causes of AF cannot be removed, and the electrical and anatomical remodeling caused by AF itself makes it difficult to reverse AF and maintain sinus rhythm. Therefore, the control of ventricular rate has become a clinical alternative [3]. LAAC prevents thrombosis in the left atrial appendage during AF, thus reducing the risk of long-term disability or death due to thromboembolism in patients with AF. Radiofrequency ablation has become the most effective method for the radical treatment of paroxysmal tachyarrhythmia. Although radiofrequency ablation can restore sinuses in more than 70% of patients with paroxysmal AF, the long-term success rate is low, and the treatment of persistent or permanent AF is limited and ineffective [4, 5]. In recent years, through a large number of clinical studies, it has been found that TCM has unique advantages in the treatment of AF, such as less side effects, low toxicity, long effective duration, and high compliance, which has played a positive role and is widely used in clinical practice.

There is no name of AF in ancient TCM books. According to clinical symptoms and signs, AF should be classified as “palpitation,” and “shocking palpitation.” Since the book of “Huangdi Neijing” was published, doctors of different generations have discussed palpitation, and a variety of prescriptions have been recorded to be used for the treatment of palpitation, such as Zhigancao Decoction, Erchen Decoction, and Taoren Honghua Decoction. It is found that TCM has a certain curative effect in reducing ventricular rate, increasing cardioversion rate and improving cardiac function and its clinical symptoms [6]. Lin Zhongxiang, a professor of Shanghai Traditional Chinese Medicine in Longhua Hospital, has been engaged in various cardiovascular diseases including arrhythmia and AF for a long time. He has accumulated rich experience in clinical practice. The prescription of “Huatan Dingji Decoction”(HTDJ) for resolving phlegm and resolving palpitation is a prescription for the main syndromes of qi deficiency and phlegm opacities created by Tangnuo, one of his students, according to Professor LinZhongxiang’s academic idea, and combined with long-term clinical treatment experience. The whole recipe is composed of pinellia ginger, tangerine peel, astragalus root, atractylode rhizome, Poria, Coptis chinensis, cinnamon, keel, oyster, Radix ophiopogonis, Chinese date, Epimedium, and sophora flavescens. The prescription has a definite effect on the treatment of heart palpitation. A large number of related patients have taken this prescription for a long time, but it is limited to individual clinical cases and lacks a relevant evidence-based summary. Therefore, the purpose of this study was to evaluate the efficacy and safety of “HTDJ” in the treatment of AF with western medicine, to provide evidence-based medical evidence of short-term and medium-term clinical efficacy of integrated Chinese and Western medicine in the treatment of AF on the basis of TCM syndrome differentiation, and to lay a foundation for further clinical development and application.

## Methods/design

### Study objectives

The purpose of the experiment is to evaluate the efficacy and safety of HTDJ plus western medicine in the treatment of atrial fibrillation, provide medical evidence of short-term and medium-term clinical efficacy for the treatment of atrial fibrillation with integrated traditional and western medicine on the basis of TCM syndrome differentiation, and lay the foundation for further clinical development and application.

### Study design and settings

**Fig. 1:**
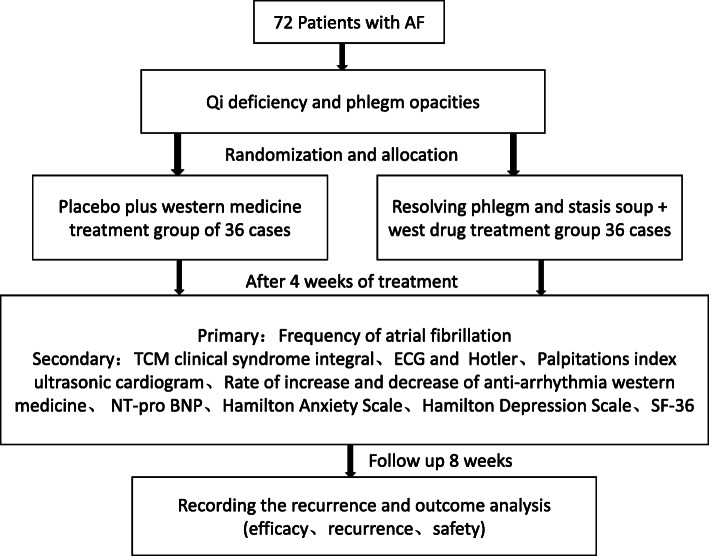
Trial flow chart

We designed this study as a randomized, double-blind, single-simulated placebo-controlled clinical trial. The study will be conducted in the Department of Cardiology, Longhua Hospital, Shanghai University of Traditional Chinese Medicine, to achieve the goal of 76 participants with atrial fibrillation (qi deficiency and phlegm opacities) in total. The participants who fulfill both the inclusion and exclusion criteria will be recruited into the study. After participants signed the consent form, they will be randomly assigned to the treatment or control group. Participants in the treatment group will receive HTDJ, whereas those in the control group will receive placebo granules. The participants will continue to receive systemic therapy, as judged by their treating physician. All changes in symptoms, prescriptions, relevant scores, and macroscopic characteristics (based on photographic evidence), along with any adverse events (AEs), will be recorded. The experimental design is shown in Fig. 1.

### Eligibility criteria

Eligible patients will be those who fulfill all of the following inclusion criteria and who do not have any of the listed exclusion criteria. The planned enrollment period is 12 weeks.

### Diagnostic criteria

The diagnosis of atrial fibrillation can be confirmed based on clinical manifestations, physical examination, and electrocardiographic characteristics as follows [7]: (1) clinical manifestations: palpitation, chest tightness, and decreased exercise tolerance; (2) physical examination: heart auscultation varies in the heart rate, and heart sounds vary in strength, rhythm is absolutely irregular, and pulse is short; and (3) electrocardiogram: *P* wave disappear, the *f* wave replaces it, the frequency is about 350–600 times, and the QRS complex rhythm is absolutely irregular.

### Inclusion criteria

The inclusion criteria are as follows: (1) The patient’s age is 18–85 years; (2) atrial fibrillation, seizure frequency ≥2 times a month (at least one ECG diagnosis); (3) the main syndrome of TCM syndrome differentiation was Qi deficiency-caused phlegm stagnation; and (4) have a correct understanding of the significance of the study, have good compliance with the observation and evaluation of the researcher, and voluntarily accept the clinical trial and fill in the clinical consent form.

### Exclusion criteria

The exclusion criteria are as follows: (1) patients with primary diseases such as severe lung, liver, renal insufficiency, and hematopoietic system or severe cardiac dysfunction (NYHA IV grade); (2) atrial fibrillation caused by obvious incentives such as fatigue, mental stress, mood swings, drug poisoning, and electrolyte disturbance; (3) mental illness and poor control of the condition; (4) patients whose heart rate is less than 50 beats per minute (such as sick sinus syndrome, atrioventricular, or intraventricular block, who intend to install a pacemaker); (5) pregnant or lactating women; (6) cachexia in the terminal stage of malignant tumor; and (7) for various reasons, the efficacy cannot be determined or the data is incomplete.

### Who will take informed consent?

A study investigator or medical staff member that has received adequate training will provide the participant with extensive explanations. Upon agreeing to provide consent, the participant will be invited to sign and date the informed consent form, at which time a participant identification (PID) number will be assigned to facilitate PID throughout the study.

### Additional consent provisions for collection and use of participant data and biological specimens

Eligible participants will be invited to sign the written informed consent regarding participation in the trial (procedures, risks, options for dropping out), regarding the use of laboratory data, and regarding the collection, storage, and use of biological specimens.

### Randomization

This study uses a center-based stratification and block randomization method. The randomization sequence will be generated by study investigators who are statisticians. Patients will be allocated in a 1:1 ratio, aiming to balance baseline characteristics between the groups. Participants will be assigned a PID number, which will be used for subject identification throughout the study. Information regarding the random-number block will be delivered to the participating centers along with the intervention drugs.

### Double-blind

The study is designed as a double-blind investigation. The participants, study monitors, and study investigators will be blinded throughout the duration of the study. The PID will be the only information linked to group allocation. Random codes will be maintained by Cui Xuejun, associate researcher, and director of the Office of National Traditional Chinese Medicine Clinical Research Base of Longhua Hospital to ensure concealment.

### Interventions

Eligible patients will be randomized to either the treatment group or the control group. The treatment group was treated with HTDJ plus western medicine. The control group was treated with placebo granules plus western medicine. Western medicine includes cardioversion drugs and heart rate control drugs, as well as myocardial energy metabolism drugs, must be in keeping with “the 2015 Atrial Fibrillation: Current Understanding and Treatment Recommendations.” The HTDJ and placebo were produced and packed in a single batch (production batch number: HTDJ: 1070649; Placebo: 1070649) by Shanghai Wan Shicheng Pharmaceutical Co., Ltd, which has no conflicts of interest relevant to this study. The test results of drug quality were consistent with the Chinese Medicine Standards of the State Food and Drug Administration. The placebo is composed of 10% crude HTDJ and 90% starch, which have the same appearance and scent as the active treatment drugs. Participants will take one bag twice a day for 4 weeks, while avoiding oral administration of other Traditional Chinese Medicine for efficacy evaluation. Then, do not accept Traditional Chinese Medicine, only western medicine treatment, followed up for 8 weeks, another clinical evaluation.

### Outcomes

#### Primary outcome

- Frequency of atrial fibrillation attacks (from baseline to weeks 4, 8, and 12)
  - ° Significant effects: The frequency of attacks decreased by more than 70%.
  - ° Effective: The frequency of attacks decreased between 30% and 70%.
  - ° Invalid: The frequency of attacks decreased by less than 30%.
  - ° Deterioration: Increased attack frequency.

#### Secondary outcomes

- Echocardiography (from baseline to weeks 4 and 12): Echocardiographic measurement of LVEF, SV, left ventricular end-diastolic diameter (LVEDD), left ventricular end-systolic diameter (LVESD), ratio of left ventricular early diastolic fast filling peak to late diastolic filling peak (E/a), and ratio of early diastolic velocity of mitral valve to early diastolic annular velocity (E/e).
- Hamilton Anxiety Scale, Hamilton Depression Scale (from baseline to weeks 4, 8, and 12). HAMA is mainly used to assess the severity of patients’ anxiety symptoms, generally more than 14 points considered meaningful. HAMD is one of the most commonly used scales to assess depression in clinical practice, and more than 20 points are considered meaningful.
- Rate of increase and decrease of anti-arrhythmia western medicine (from baseline to weeks 4, 8, and 12).
- SF-36 (from baseline to weeks 4, 8, and 12). The MOS Item Short from Health Survey (SF-36) was used to evaluate the quality of life of patients.
- NT-proBNP (from baseline to weeks 4 and12): NT-proBNP is a reliable and sensitive indicator of heart failure. One of the causes of heart failure is atrial fibrillation. Its elevation is positively correlated with the severity of heart failure. Changes in the levels of NT-proBNP in each group will be measured before and after treatment.
- Integral TCM syndrome score (from baseline to weeks 4 and 12; the scores and details are presented in Tables 1 and 2): A scale of 0–6 points and half quantitative points will be used to score the patients according to the severity of clinical symptoms [7].
  - ° Significant effect: inhibition or complete suppression of primary and secondary clinical symptoms and reduction of syndrome score by ≥ 70%
  - ° Effective: significant improvement in clinical symptoms, and reduction of syndrome score by 30–70%
  - ° Ineffective: reduction of treatment syndrome score by < 30%
  - ° Aggravation: the score after treatment exceeds the score before treatment

**Table 1:** Traditional Chinese Medicine symptom score

	None	Mild	Moderate	Severe
Palpitation	0	2	4	6
Panting	0	2	4	6
Lassitude	0	2	4	6
Expectoration	0	2	4	6
Weight	0	2	4	6
Tongue fur and type of pulse	0	2	4	6

**Table 2:** Detailed description of symptoms according to Traditional Chinese Medicine

	None	Mild	Moderate	Severe
Palpitation	No symptom	Occasional occurrence; slight uncomfortable feeling	Regular occurrence; lasts for a long duration; intense uncomfortable feeling	Frequent occurrence; uncontrolled; Considerable influence on the quality of life
Panting	No symptom	Slight and no influence on routine activities	Heavy, but can still manage to perform routine activities	Too heavy, cannot perform routine activities
Lassitude	No symptom	Slight and can work	Heavy, but can still manage to work	Too heavy, cannot work or continue routine activities
Expectoration	No symptom	Less sputum	Moderate amounts of sputum	A lot of phlegm
Weight	BMI:18.5-24	BMI:24-27	BMI:27-30	BMI≥30
Tongue fur	Thin white tongue	Tongue coating is thin and yellow	Thin glossy coating of the tongue	Moss greasy thick

### Safety monitoring

Routine blood test, liver and kidney function test, electrolytes test, and urine test (from baseline to weeks 4) are the basic indicators that reflect the general situation and basic safety evaluation.

### Criteria for discontinuing or modifying allocated interventions

The criteria for discontinuation are as follows: (1) In case of serious adverse events, the clinical experimenter should be stopped according to the doctor’s judgment. (2) If the disease worsens in the course of the disease, or there are other conditions affecting the observation of the study, the clinical experimenter should be stopped and invalid cases should be treated according to the doctor's judgment. (3) Important deviations occurred in the implementation of clinical trial protocols, such as poor compliance, making it difficult to evaluate drug effects. (4) The subject is unwilling to continue the clinical trial during the clinical trial and proposes to the doctor in charge to withdraw from the clinical trial.

For these cases, the reasons for discontinuation shall be recorded in detail. If there is a review, the results of the last major efficacy test shall be taken as the final results for statistical analysis, and the CRF table and REDCap (Research Electronic Data Capture) shall be kept for future reference.

### Strategies to improve adherence to interventions

The drug quantity and date of drug intake by each patient will be recorded. The patients will be required to return any unused drugs during each visit, which will be recorded by the researchers.

### Observation records of adverse events

During the trial period, the adverse event record form should be filled in truthfully. The occurrence, severity, duration, measures taken, and outcome of the adverse event should be recorded. Adverse events should be recorded in the designated clinical case observation form (CRF) adverse event form. The person uses mild, moderate, and severe to describe the intensity of the adverse event and evaluates the possible association between the adverse event and the study drug and combination drug to determine whether it is an adverse reaction. When adverse events are found, the researchers will contact the project leader immediately and start the emergency treatment plan. In case of serious adverse events, the principle of “free treatment first” shall be implemented, and the report shall be reported to the organizer and the ethics committee within 24 h, and the “Serious Adverse Events Report Form” shall be filled out and signed and marked with the date. According to the severity of adverse reactions, the follow-up visit can be in hospital, outpatient, home visit, telephone, communication, and other forms.

### Research process record points

The subject content and data at each time point, according to the patient’s hospitalization period, will be recorded as shown in Table 1. Specifically, the screening period (−3~0 days) will be 0 to 3 days before recruitment. The 4-week treatment period was planned in two cycles of 2 weeks, with a return visit between cycles to check for AEs and monitor compliance. All interventions will be stopped after 4 weeks. Follow-up will be conducted on weeks 4 and 12. The detailed information is shown in Table 3.

**Table 3:** Research flowchart

Activity	Phase(time points)	Screening stage	baseline period	intervention	Follow-up	
		−3d~0d	0d	4w	8w	12w
**Enrolment**	Sign informed consent	✔				
	Inclusion and exclusion criteria	✔				
	Demographic data	✔				
	Medical history and treatment history	✔				
	Basic data collection	✔				
**Outcome assessment**	Frequency of atrial fibrillation		✔	✔	✔	✔
	ECG and Holter		✔	✔	✔	✔
	Ultrasonic cardiogram		✔	✔	✔	✔
	TCM clinical syndrome integral		✔	✔	✔	✔
	Rate of increase and decrease of anti-arrhythmia western medicine		✔	✔	✔	✔
	NT-proBNP		✔			✔
	Hamilton Anxiety Scale		✔	✔	✔	✔
	Hamilton Depression Scale		✔	✔	✔	✔
	SF-36		✔	✔	✔	✔
	PVO		✔	✔	✔	✔
	Anaerobic threshold		✔	✔	✔	✔
**Safety assessment**	Physical examination		✔	✔	✔	✔
	Blood routine examination		✔	✔		
	Urine routine and urinary sediment		✔	✔		
	Liver function		✔	✔		
	Renal function		✔	✔		
	Blood lipid		✔	✔		
	GLU		✔	✔		
	INR		✔	✔		
	CHA2DS2-VAS		✔	✔		
	HAS-BCED		✔	✔		
**Other information**	Randomization	✔				
	Hemorrhage		✔	✔	✔	✔
	Readmission		✔	✔	✔	✔

### Sample size

The sample size of this study was calculated based on the analysis of data published in previous articles on the treatment of atrial fibrillation by Chinese medicine and the expected value of efficacy. It was assumed that the total efficacy rates in the study and control groups were 91.1% and 53.3%, respectively [8]. The set *α* = 0.025, *β* = 0.2, superiority limit value*δ*= 10%, and the sample size of each group will be calculated as 30 cases, considering a 20% loss to follow-up, that is, a total of 76 cases.

### Recruitment

Participants will be recruited from the Longhua Hospital affiliated to Shanghai University of Traditional Chinese Medicine by the researchers who have a Good Clinical Practice certificate. The patients will be informed about the objectives, approaches, potential adverse effects, and advantages of this trial. The planned enrollment period is 21 weeks.

We will use three main ways to ensure enough participants in our project:

1. Posting recruitment information in the hospital area (such as outpatient lobby and ward door);
2. Posting recruitment information on popular communication platforms such as WeChat public account and hospital service account;
3. If potential participants are found in the outpatient and inpatient staff of the Longhua Hospital, the researchers will ask the patients if they are willing to participate

### Confidentiality

Only the investigator participating in the research clinical trial will have access to the subject’s personal medical records, and they will sign an “Investigator statement” or “Confidentiality Commitment” that includes confidentiality. Data will be processed in a “data anonymity” manner, and the information that can identify the individual identity of the subject will be omitted.

### Statistical analysis

All data are entered using excel worksheets, and SPSS22.0 statistical software is used for statistical analysis. For measurement data, *t* tests are performed for those that conform to the normal distribution, and the data are represented by the mean ± standard deviation; the row-rank conversion that does not conform to the normal distribution For non-parametric test, the data are represented by M (QR). The constituent ratio data uses the *χ*^2^ test. The one-way ordered data uses the rank sum test. *P*>0.05 is not statistically significant, and *P*<0.05 is statistically significant, two-sided test.

To further clarify the research conclusions of this clinical trial, we will perform subgroup analysis by gender or age in the future.

### Composition of the coordinating center and trial steering committee

The coordinating center is the Department of Cardiology of Longhua Hospital Affiliated to the Shanghai University of Traditional Chinese Medicine and the principal investigator, and it consists of the principal investigator(PI), clinical research coordinator(CRC), full-time data manager, and project quality control experts. The trial steering committee is the Good Clinical Practice Office of Longhua Hospital, affiliated with the Shanghai University of Traditional Chinese Medicine. The responsibilities include agreement and amends to the protocol, receiving reports from the coordinating center, supervising the trial, reviewing trial progress, and reviewing the paper for publication. Meetings are conducted every 6 months.

### Data management and monitoring

In this study, the CRF will be filled out by the researchers in a timely manner. Moreover, we will also use the REDCap data platform. Study data were collected and managed using REDCap electronic data capture tools hosted at LongHua Hospital Shanghai University of Traditional Chinese Medicine. REDCap is a secure, web-based software platform designed to support data capture for research studies.

To ensure confidence in the data, data monitoring will be conducted by the data monitoring committee (DMC). The data monitoring committee (DMC) includes a researcher, who will not be involved in the data collection, data manager, biostatistician, and ethicist (independent from the sponsor and free from competing interests). The DMC will conduct periodic interim evaluations and discontinue the trial if there are obvious differences between the two groups. To ensure confidence in the data, study-related files including consent forms, the CRF, questionnaires, medical records, and other records will be stored in a locked space or on a password-protected computer in each hospital for 5 years after study completion. Data monitoring will be conducted by the data and safety monitoring committee. Auditing can be conducted to this trial by the quality assurance team at the supporting institution, independent from the investigators. The project management group will meet to review trial conduct every 2 weeks, and the trial steering group, DMC, and ethics committee will meet to review trial conduct every 6 months.

### Plans to promote participant retention and complete follow-up

All researchers treat the participants in a friendly and respectful way, answering all questions they may have while encouraging them to participate—economic compensation including transportation, food, and communication reimbursement. In addition, all of the participants will be compensated 100 Chinese yuan. The researchers will send reminders to the patients 2 to 3 days before each follow-up.

### Interim analyses

We started performing statistical analysis when the samples reached 44. After the interim results are obtained, the primary investigator will determine whether the experiment will be continued.

### Dissemination plans

The final data will be submitted to the Shanghai Science and Technology Committee in the form of a research report by the researchers after completion of the study. The results will be published in a peer-reviewed academic journal to share the findings with the general public and healthcare professionals.

## Discussion

As the most common arrhythmia, AF is characterized by palpitations, chest tightness, shortness of breath, dizziness, and fatigue. It has serious complications and seriously affects people’s quality of life and health. At present, the treatment of AF is mainly treated by western medicine, mainly through drugs and surgery, but anti-arrhythmic drugs have side effects. The effect of radiofrequency ablation is unstable and easy to relapse. In recent years, through a large number of clinical studies, it has been found that the treatment of AF with TCM has certain efficacy in reducing the ventricular rate, improving the recovery rate, and improving the cardiac function and clinical symptoms. At present, Wenxin granule and Shensong Yangxin capsule on the market are mainly targeted at patients with Qi and Yin deficiency and blood stasis, and “deficiency” and “phlegm” are important pathogenesis of AF [9–12]. Due to the aging of the population and the characteristics of diet structure, clinical patients with TCM pathogenesis of “deficiency” and “phlegm” account for a certain proportion. The current western medicine and surgical treatment of patients with AF cannot fundamentally change the trend of atrial fibrosis and human body degeneration, which requires long-term drug maintenance and high recurrence rate. A long-term use of amiodarone, sotalol, and other western drugs has certain side effects and great limitations, so better treatment methods are needed.

HTDJ is designed from the pathogenesis of AF (qi deficiency and phlegm opacities). In this prescription, Huanglian Wendan Decoction can clear away heat and dampness and treat disturbance of the gastric qi. Pinellia ternata and Pericarpium Citri Reticulatae are used to regulate qi-flowing for removing the phlegm; Poria cocos can fortify the spleen and percolate dampness. Coptis chinensis can purge intense heat and detoxicate; Chinese date can benefit the spleen and stomach. LiuJunZi Decoction can replenish qi to invigorate the spleen and remove the phlegm. On the basis of Sijunzi Decoction, increasing the dosage of Atractylodes macrocephala can improve the effect of drying dampness and resolving phlegm. Coptis chinensis, cinnamon, and Jiaotai Pill can coordinate the heart and the kidney. On this basis, adding fossilizid and concha ostreae can quiet the spirit by heavy settling. Astragalus membranaceus can tonify middle-Jiao and Qi, while dwarf lilyturf tuber can nourish yin and promote the secretion of the saliva. Herba Epimedii can mildly reinforce the kidney yang and light yellow sophora root can clear away heat and dampness. The prescription can replenish qi to invigorate the spleen and remove the phlegm, nourish the heart and quiet the spirit, coordinate the heart and the kidney, and balance yin and yang.

Clinical and pharmacological studies have shown that Jiaotai Pill has therapeutic and anti-arrhythmic effects on AF [13, 14]. Pinellia ternata and its processed products are also anti-arrhythmic [15], and Pericarpium Citri Reticulatae has vasodilatory effects that help to induce an increase in coronary flow, which may have the effect of lowering blood pressure and slowing the heart rate [16]. The effects of Coptis chinensis include blood pressure lowering, antibiosis, anti-inflammatory, anti-tumor, regulating blood lipid, and anti-arrhythmia [17]. Icariin can effectively avoid the damage of myocardial cells and vascular endothelium caused by adverse factors. In addition to inhibiting apoptosis in cardiac myocytes, icariin can also effectively regulate blood lipid and protect the cardiovascular system [18]. In addition, Huanglian Wendan Decoction has a certain therapeutic effect on coronary heart disease with rapid AF [19]. Matrine has antiarrhythmic and electrophysiological effects [20]. Artemisia apiacea is the main drug for clinical treatment of arrhythmia [21], and arteannuin has electrophysiological characteristics of antiarrhythmia [22].

There are a large number of clinical effective cases of HTDJ in the treatment of atrial fibrillation, but there is still a lack of relevant evidence-based evidence summary. Therefore, this study aims to evaluate the efficacy and safety of “Huatan Dingji Prescription” plus western medicine in the treatment of AF, in order to provide preliminary data for the treatment of patients with AF. It can further provide evidence for the clinical application of TCM in the treatment of AF.

### Trial status

The trial has been registered on Chinese Clinical Trial Registry (ID: ChiCTR2000030517). The final protocol version is 2.0 and is dated January 31, 2020. Recruitment began on April 15, 2020, and recruitment will be completed on January 1, 2022.

## Data Availability

We declare that the materials described in the manuscript, including all relevant raw data, will be freely available to any scientist wishing to use them for non-commercial purposes without breaching participant confidentiality.

## Abbreviations

AF: Atrial fibrillation
HTDJ: Huatan Dingji Decoction
TCM: Traditional Chinese Medicine
HF: Heart failure
PE: Stroke, pulmonary embolism
LAAC: Left atrial appendage closure
RF: Radiofrequency ablation
PID: Participant identification
AEs: Adverse events
EF: Ejection fraction
SV: Stroke volume
LVEDD: Left ventricular end-diastolic diameter
HAMA: Hamilton anxiety scale
HAMD: Hamilton depression scale
CRF: Case observation form
DMC: Data monitoring committee
SF-36: The MOS 36-item short-form health survey
REDCap: Research Electronic Data Capture
